# Surfacing behavior and gas release of the physostome sprat (*Sprattus sprattus*) in ice-free and ice-covered waters

**DOI:** 10.1007/s00227-013-2334-1

**Published:** 2013-10-04

**Authors:** Ingrid Solberg, Stein Kaartvedt

**Affiliations:** 1 Red Sea Research Center, King Abdullah University of Science and Technology, Thuwal, 23955-6900 Saudi Arabia; 2 Department of Biosciences, University of Oslo, PO Box 1066, Blindern, 0316 Oslo, Norway

## Abstract

Upward-facing echosounders that provided continuous, long-term measurements were applied to address the surfacing behavior and gas release of the physostome sprat (*Sprattus sprattus*) throughout an entire winter in a 150-m-deep Norwegian fjord. During ice-free conditions, the sprat surfaced and released gas bubbles at night with an estimated surfacing rate of 3.5 times per fish day^−1^. The vertical swimming speeds during surfacing were considerably higher (~10 times) than during diel vertical migrations, especially when returning from the surface, and particularly when the fjord was not ice covered. The sprat released gas a few hours after surfacing, suggesting that the sprat gulped atmospheric air during its excursions to the surface. While the surface activity increased after the fjord became ice covered, the records of gas release decreased sharply. The under-ice fish then displayed a behavior interpreted as “searching for the surface” by repeatedly ascending toward the ice, apparently with limited success of filling the swim bladder. This interpretation was supported by lower acoustic target strength in ice-covered waters. The frequent surfacing behavior demonstrated in this study indicates that gulping of atmospheric air is an important element in the life of sprat. While at least part of the population endured overwintering in the ice-covered habitat, ice covering may constrain those physostome fishes that lack a gas-generating gland in ways that remain to be established.

## Introduction

The clupeid sprat (*Sprattus sprattus*) is a physostome fish with an open swim bladder. Clupeids have one pneumatic duct connecting the swim bladder to the stomach and one anal duct leading from the posterior swim bladder to the vent (Blaxter et al. 1979). In comparison with physoclists that can regulate their buoyancy by gas glands in their swim bladder, most physostomes are without any gas secretion organs (Blaxter et al. 1979) and seem to attain gas for refilling their swim bladder by “gulping” atmospheric air at the surface (Brawn 1962; Blaxter and Hunter 1982; Blaxter and Batty 1984). Blaxter and Batty (1984) showed that herring (*Clupea harengus*) with artificially emptied swim bladders could refill them by swallowing air at the surface; meanwhile, the individuals without access to the surface suffered a high mortality.

Due to a barrier of guanine crystals in the swim bladder wall, the diffusion rates over the wall are estimated to be low in herring, and the bladder can thus retain swallowed gas for several weeks or even months depending on the depth and pressure (Blaxter et al. 1979). However, clupeids may release gas from the swim bladder through their anal duct (or sometimes through the mouth; Thorne and Thomas 1990; Wahlberg and Westerberg 2003). Schools of herring have been reported making foams of bubbles at the surface as a result of air release during upward swimming (Nøttestad 1998), a phenomenon also known and used in the past among fishermen as a means of locating herring (Sundnes and Bratland 1972). Reduced pressure, stress response, or predator avoidance are some of the suggestions for the gas release behavior (Brawn 1962; Thorne and Thomas 1990; Hahn and Thomas 2008).

For all clupeids, reports on air gulping and gas release have so far been circumstantial, without any systematic assessments of behavior or timing on diel and seasonal scales associated with such events. The potential effect of ice cover is also unknown. While marine clupeids normally live in ice-free habitats, certain populations of sprat occur in habitats that may become ice covered in winter, like in the Baltic and some fjords (Ojaveer and Kalejs 2005; Casini et al. 2006; Kaartvedt et al. 2009). Also, many populations of clupeid fish live in freshwater (Janssen and Brandt 1980; Harman and Albright 2002; Cyterski and Ney 2005) where ice cover during winter is more common. However, to what extent ice cover may affect a possible gulping behavior and restrain the fish’ ability to regulate their buoyancy or other swim bladder functions remains to be examined.

The primary objective of this study was to address the surfacing and gas release behavior of sprat by the use of upward-facing echosounders that provided continuous, long-term measurements. We assess patterns in the frequency of surfacing events during an entire winter, measure vertical swimming speeds during ascent and descent to and from the surface, and assess the occurrence of gas bubble release. The fjord froze over in the course of the winter, providing the opportunity of revealing how the overwintering sprat were affected by ice cover.

## Materials and methods

The study took place in Bunnefjorden, Norway, throughout the entire overwintering period of 2009/2010. Bunnefjorden (150 m deep) is the innermost part of the Oslofjord, and due to restricted water exchange, the deep water of the fjord is normally hypoxic. Bunnefjorden often becomes ice covered during winter. A map depicting the study area and the location of the echosounders (59.79° N, 10.72° E) is given in Klevjer and Kaartvedt (2011).

### Field campaigns

To assess environmental parameters and sampling of acoustic targets, field campaigns were carried out using the research vessel of the University of Oslo, “Trygve Braarud”. Salinity and temperature were measured by a Falmouth Scientific Instruments CTD equipped with Niskin bottles. Water from these bottles was later analyzed for oxygen content using the standard Winkler method. Fish were sampled in a total of 33 pelagic hauls (both diurnal and nocturnal) carried out during two sampling days in December 2009 and one sampling day in April 2010. The pelagic trawl has an aperture of about 100 m^2^ and was towed at two knots. The mesh size is 20 cm near the opening, declining to around 1 cm at the rear part and 3 mm in the cod-end. A Scanmar depth sensor was connected to the trawl, measuring the trawl depth during sampling. The trawl was equipped with a multisampler codend (Engås et al. 1997) enabling vertically stratified sampling.

### Continuous acoustic studies

Three submerged echosounders kept in pressure proof casings were deployed from November 11, 2009 until April 12, 2010. A Simrad EK60 38 kHz echosounder (7° beam width) was deployed at the bottom of the fjord (150 m), and adjacent buoys with echosounders floating at 80 m (Simrad EK60 120 kHz, 7° beam width) and 30 m (Simrad EK60 200 kHz, 7° beam width) were deployed for enhanced resolution in shallow waters. The upward-facing transducers were mounted in steel frames with gimbal couplings to ensure horizontal orientation of their surfaces. The temporal resolution of data (the ping rate) was set to 1–2 ping s^−1^ (38 kHz), 2 ping s^−1^ (120 kHz), and 2.5–6 ping s^−1^ (200 kHz). The echosounders were connected to land with cables that provided electricity and transmitted data to a pc on shore. All raw data were stored for later analyses.

We here use data from the bottom-mounted echosounder for abundance estimates (echointegration) and also present echograms from this echosounder to illustrate the population distribution and behavior. Individual surfacing and gas release behavior, and vertical swimming speed are analyzed based on data from the shallowest echosounder that provided superior resolution of near-surface waters. Data from the echosounder at 80 m (120 kHz) were used as a supplement to assess the amount and depth of gas releases that may have occurred below the shallowest echosounder.

### Acoustic post-processing

Acoustic data from 200 kHz (November 11–December 12, 2009; January 01–March 31, 2010; and April 05–12, 2010) were processed in the Sonar_5 Pro software program (Balk and Lindem 2005). Target tracking (TT), which allocates subsequent echoes to the same target, was used to assess acoustic size [target strength (TS)] and swimming speed of surfacing fish. The fish were identified as sprat based on trawl results from the present and previous studies in the fjord (Solberg et al. 2012) and on comparison of TS measurements of sprat (Røstad 2006).

**Fig. 1:**
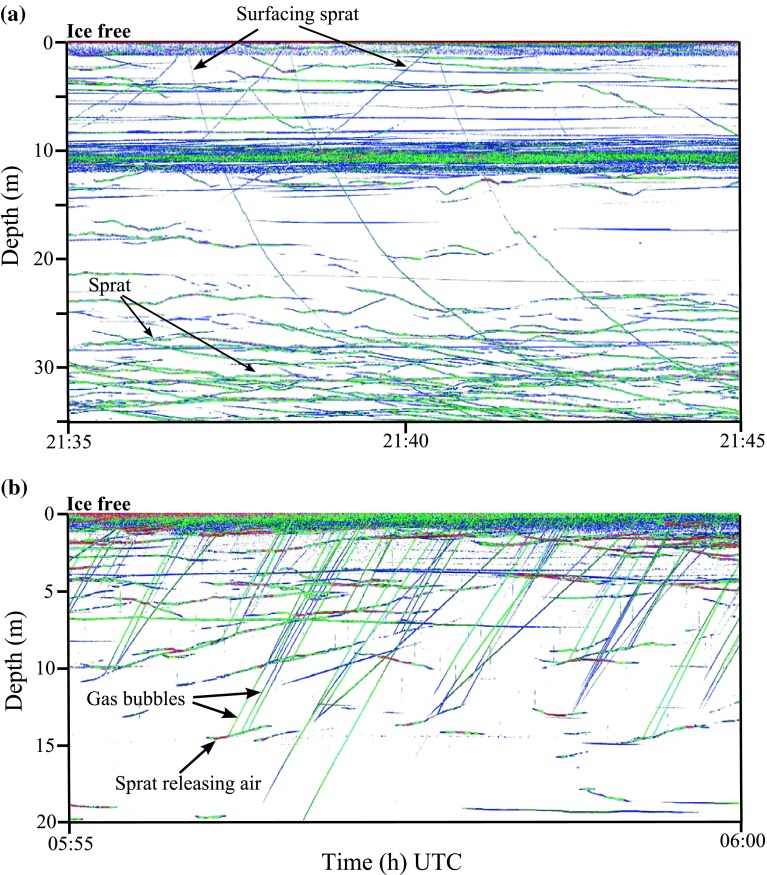
**a** Acoustic record (120 kHz) from November 11, 2009, showing sprat surfacing before migrating ~30 m to join deeper distributed fish. **b** Acoustic record (200 kHz) from November 12, 2009, showing sprat releasing gas (every *line* represents one burst of *bubbles*). The strong echoes of *horizontal lines* in echogram **a** are backscatter caused by non-biological noise (~10 m)

Surfacing fish were detected by manual target tracking (TT) where echo traces from single organisms are selected from the echogram and combined into tracks by the researcher. The data were tracked in the single echo detection (SED) echogram. This only displays a subset of the data that fulfill criteria for a well-defined single target, in contrast to the ordinary amplitude (Amp) echogram that shows all backscatter. The SED threshold was set to −70 dB. Any tracks with TS stronger than −45 dB were removed during post-processing (to sort out fish larger than sprat). The tracking was done between the surface and the upper 15–20 m of the water column. The surface area of the beam was 10.5 m^2^. Surfacing sprat could easily be distinguished from other echoes as they were seen as thin lines rising up to the surface before quickly returning to depth (see Amp-echogram example Fig. 1a). Only traces that were both clearly ascending to and descending from the surface were selected as a single “surfacing event.” Ascending and descending trajectories were tracked separately in order to measure upward and downward swimming speed for each surface excursion. Statistical analyses of swimming speed and TS measurements were performed using two-way ANOVA and post hoc Tukey’s test implemented in the software R.

Released gas could be seen on the echogram as a thin line ascending from the fish up to the surface (see Amp-echogram example Fig. 1b). Every “gas release event” was registered using a “mouse tip monitor” in Sonar_5 (by double clicking with the mouse, the time of the released gas was recorded). The files were manually checked for gas releases in the Amp-echogram with a threshold of −75 dB. In some cases, traces of ascending bubbles were observed without any connection to a fish. These trajectories were not included. Such traces likely arise from fish outside the narrow acoustic beam close to the transducer or below the buoy of the echosounder when releasing air. By excluding these, the total number of gas releases will be underestimated. Nevertheless, since it was known that gas bubbles due to metal corrosion sporadically rose from the steel box of the echosounder, this method was considered most appropriate.

### Biomass integration

To relate the frequency of surfacing to abundance of fish, the abundance of sprat was assessed throughout the entire winter. The nautical area scattering coefficient (s_A_) was determined by integration of 38 kHz acoustic data from 0 to 100 m depth, using the LSSS software (Korneliussen et al. 2006). The depth range below 100 m was excluded due to noise from non-biological sources. This is not a major problem because the water column was devoid of biological backscatter below 100 m during the first half of the winter (November to mid-February) due to anoxic conditions, and only a minor part of the sprat population stayed deeper than 100 m after an influx of more oxygenated water in mid-February (acoustic results, not shown).

Integration was made over 5-m depth intervals and 30 min periods from November 11, 2009 to April 12, 2010 at a threshold of −73 dB. As backscatter ascribed to krill was nearly absent this year, this threshold could be used without smaller organisms affecting the results. All acoustic backscatter was allocated to sprat and results are presented as daily total area backscattering coefficient (s_a_) (surface integrated, over the whole water column). The average number of sprat per m^2^ per day was calculated by dividing the backscatter value with the TS value of sprat (−48.2 dB) based on manual tracking of 38 kHz acoustic data (Røstad 2006). The average number of daily surfacing events per m^2^ was further estimated in order to calculate the average rate of surfacing per fish per day. The average number of gas release per fish per day was estimated correspondingly.

### Meteorological data

Weather data from a nearby station in Oslo (Blindern) were obtained from the climate database of the Norwegian Meteorological Institute available at (www.eklima.no). Average cloud cover per day, measured in octas, was used to map the occurrence of clear sky and overcast weather during the study period. A Web camera providing images every hour was used to monitor the ice conditions of the fjord. The fjord became ice covered in January this winter.

## Results

### Hydrography and trawl catches

**Fig. 2:**
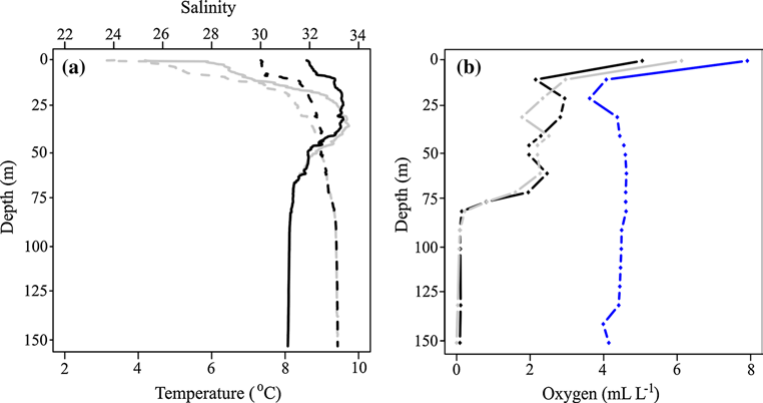
**a** Vertical profiles of salinity (*dotted lines*) and temperature (*solid lines*) in Bunnefjorden in November 2009 (*black*) and December 2009 (*gray*). **b** Vertical profiles of oxygen concentrations in Bunnefjorden during the winter 2009/2010. November (*black*), December (*gray*), and April (*blue*)

In November and December, the temperature was 8.5 and 4.5 °C in surface waters, respectively, increasing to nearly 10 °C at around 30 m. From 60 m and below, the values stabilized at around 8 °C (Fig. 2a). The salinity at the surface was 30 in November and 24 in December, the salinity increased to nearly 32 at 30 m (in both months), and in the lower half of the water column, the salinity was stable at 33 (Fig. 2a). The oxygen content in November and December 2009 ranged between 2 and 3 ml O_2_ L^−1^ from 15 to 60 m. The values decreased rapidly below 70 m, and deeper than 80 m, the values were close to 0 (Fig. 2b). A water renewal in mid-February brought more oxygenated water into the fjord, and in April, the oxygen values had increased to >4 ml L^−1^ from 30 m and deeper (Fig. 2b). The fjord became ice covered between 06 January and 08 January, after which no sampling was carried out until after the ice melted (between 01 April and 04 April).

A total of 7,345 sprat were caught during the sampling in December 2009 and April 2010, making sprat the most abundant species in the trawl catches. In December, the highest catches of sprat were obtained in the range of 60–80 m during the day (~720 sprat per 10 min of trawling) and 40–70 m at night (~85 sprat per 10 min of trawling). There were no catches of sprat below 80 m in the beginning of the winter. During daytime in April, most of the sprat were collected from 70 to 95 m (~520 sprat per 10 min) with nearly none distributed deeper than 100 m or shallower than 60 m (<5 sprat per 10 min). The catches contained furthermore a total of 162 herring (*Clupea harengus*), 58 gobiids (Gobiidae spp.), 12 whitings (*Merlangius merlangus*), 4 anchovies (*Engraulis encrasicolus*), 1 pollack (*Pollachius pollachius*), and some gelatinous plankton. Catches of other invertebrates like krill and shrimps were negligible this winter.

### Vertical distribution of sprat

**Fig. 3:**
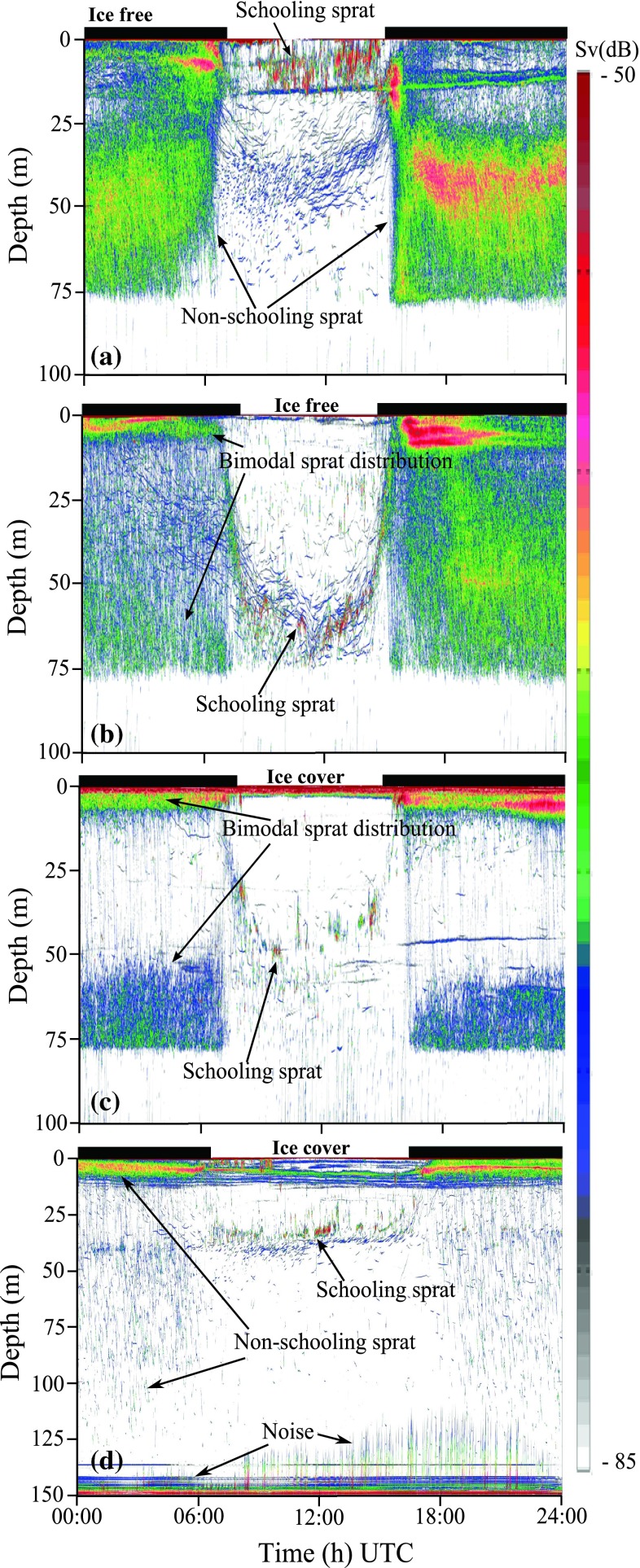
Acoustic records (38 kHz) showing vertical distribution of sprat throughout 24 h (hours of darkness are marked with *black lines* on top of the echograms). Local time is UTC + 1 h. **a** Echogram from November 14, 2009, showing schools of sprat close to the surface during daytime and the majority of the population distributed in a dispersed layer below 30 m at night. **b** December 07, 2009, the majority of sprat schools at ~60 m during the day and has a bimodal vertical distribution at night. **c** January 16, 2010, a distinct bimodal distribution is present at night with one group of sprat close to the surface and one group below 50 m. There is no acoustic backscatter in the severely hypoxic waters below ~75 m in November, December, and January. **d** February 23, 2010, a small part of the sprat population is distributed deeper than 100 m at night

The vertical distribution of the sprat population changed throughout the winter. In the beginning of November, a major part of the population was schooling in upper waters during the day, while schools dispersed and individuals descended to deeper waters at night (i.e., their behavior can be classified as inverse diel vertical migration; IDVM). Most fish were distributed in a layer ranging from around 30–60 m at night (Fig. 3a). Fish joined this layer after their excursions to the surface (Fig. 1a).

The nocturnal layer became more dispersed and extended to shallower depths by the end of the month, and by December, a part of the population carried out normal DVM and schooled in deep waters during the day. A bimodal distribution was displayed at night (Fig. 3b). A similar pattern was observed in January (when the fjord became ice covered), but the two nocturnal modes were separated over a larger depth range as one layer was distributed close to the surface and the other below 50 m (Fig. 3c). The sprat inhabited waters down to ~75 m from November until end of January. In the following months, the nocturnal distribution extended deeper, but the majority of the sprat were still distributed in the upper half of the water column (Fig. 3d). During daytime, the sprat schooled at various depths from 0 to ~90 m from February until the end of the study in April.

### Abundance of sprat

**Fig. 4:**
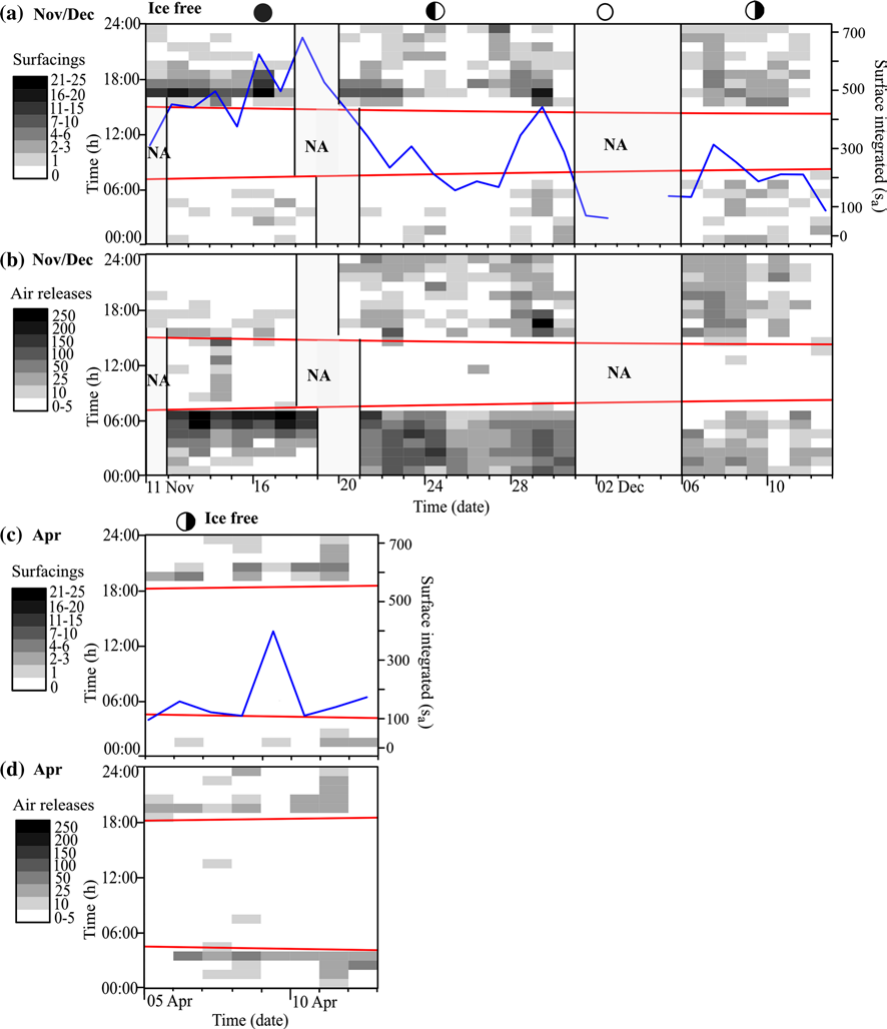
Surfacing events (**a** and **c**) and gas releases (**b** and **d**) plotted per day (*x-axis*) against hour of the day (*y-axis*) during the period November 11–December 12, 2009 (*upper*) and April 05–12 (*lower*). The *gray scaled color* bricks range over different intervals where *white* bricks represent the minimum number of surfacing events (zero) and gas releases per hour (0–5), and *black* bricks represent the maximum number of surfacing events (21–25) and gas releases per hour (201–250). Local time is UTC + 1 h (November and December) and UTC + 2 h (April). The *red lines* mark sunrise (*lower*) and sunset (*upper*), and the *blue lines* represent the surface integrated s_a_ value (biomass index). *Moon phases* are shown above the plots. *NA* indicates missing data

**Fig. 5:**
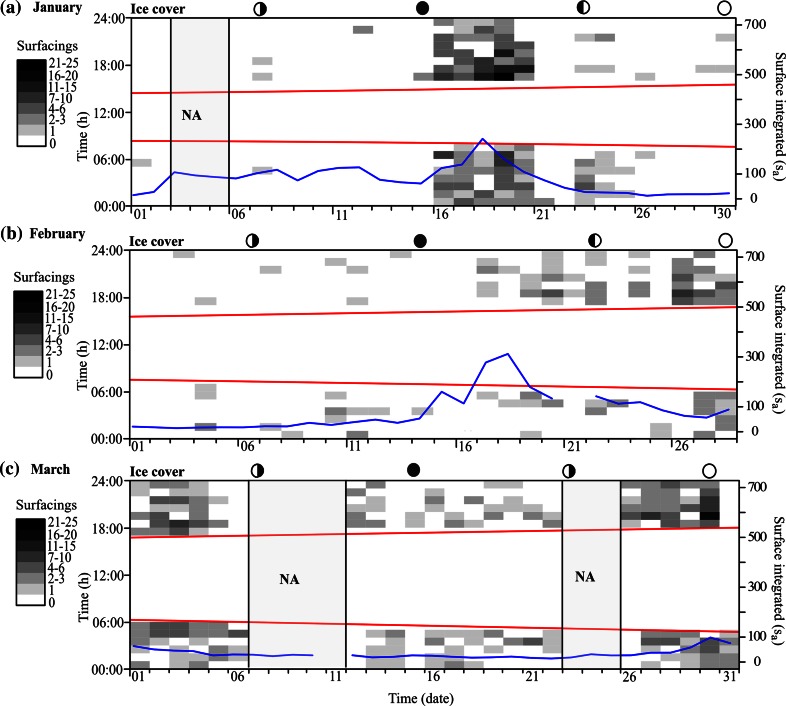
Surfacing events per day (*x-axis*) plotted against hour of the day (*y-axis*) during the ice-covered period of the winter (January 2010 (**a**), February 2010 (**b**), and March 2010 (**c**)). The *gray scaled color* bricks range over different intervals where *white bricks* represent minimum (zero), and *black* represent maximum number of surfacing events per hour (21–25). Local time is UTC + 1 h. The *red lines* mark sunrise (*lower*) and sunset (*upper*), and the *blue lines* represent the surface integrated s_a_ value (*biomass index*). *Moon phases* are shown above the plots. *NA* indicates missing data

The surface integrated values, s_a_ (interpreted as acoustic biomass), were highest in the period before the fjord froze over with a monthly average of >300 in November and ~200 in December (Fig. 4a). The monthly average values declined to <~100 during the period with ice-covered waters (January, February, and March; Fig. 5). The s_a_ increased in April (when the ice had melted) with daily values fluctuating between 100 and 400 (Fig. 4c).

### Surfacing and gas release in ice-free waters

Surfacing sprat was detected every date during ice-free conditions (November 11–December 12, 2009 and April 5–12, 2010; Fig. 4a, c). Recorded surfacing events ranged from 1 per night to nearly 50 per night, with an average daily estimated “surfacing rate” of ~3.5 times fish^−1^. There was a significant relation between the total area backscattering coefficient (s_a_) and number of surfacing fish per day (Linear regression, *r* ^2^ = 0.38, *F*_1,31_ = 20.9, *P* < 0.0000). There were no records of surfacing sprat during daytime.

**Fig. 6:**
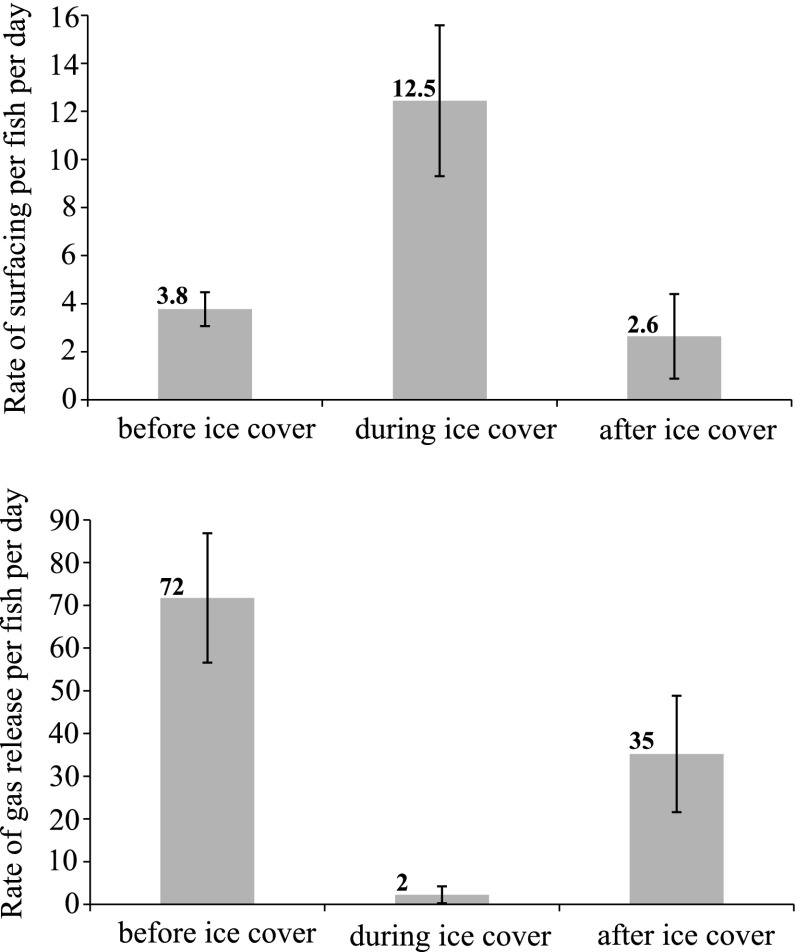
Barplot showing the average rate of surfacing per fish per day (*upper*) and the average rate of gas release per fish per day during the period before the fjord became ice covered, during ice coverage, and after the ice had melted. The *error bars* depict 95 % confidence interval

Sprat surfaced after sunset, particularly early at night, and released gas in the following hours during nighttime (Fig. 4b, d). Release of gas was prominent in the morning hours, especially in November when most of the gas bubbles were detected within 2 hours prior to sunrise (Fig. 4b). The estimated “rate of gas release” was 72 times fish^−1^ day^−1^ in the period before ice cover (November and December) and 35 times fish^−1^ day^−1^ in the period after the ice had melted, i.e., the rate of gas release was an order of magnitude higher than the rate of surfacing (Fig. 6). The relation between number of surfacing events and amount of gas release per day was significant for all months combined, but the percentage of explained variation was low (Linear regression, *r* ^2^ = 0.31, *F*_1,30_ = 14.7, *P* = 0.0006). The low relationship may to some extent be explained by fish releasing air also below the depths of our records with the 200 kHz echosounder as data from the 120 kHz echosounder showed that the fish released air between 0 and 40 m depths. However, the majority of gas release appeared to take place in the 30 m interval covered by the 200 kHz echosounder.

### Surfacing and gas release in ice-covered waters

**Fig. 7:**
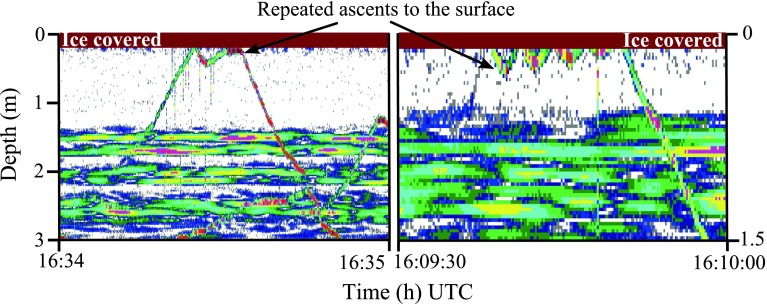
Acoustic record (200 kHz) when the fjord was ice covered in January 2010 showing two examples of sprat that repeatedly ascend to the surface before swimming down. (Such sequences of repeated ascents in between the final descent were counted as *one* gulping event). The strong echoes of *horizontal lines* are backscatter caused by non-biological noise

The fjord became ice covered between 06 January and 08 January. There were few surfacing fish during the first 2 weeks of January (<10). This was followed by a conspicuous increase in recorded surface events during the third week of January ranging from 39 to 101 per day (Fig. 5a) giving an average daily surfacing rate of ~24 times fish^−1^ for that period. The high number of surfacing fish was only apparent for around 1 week followed by a period with a few detections until the second half of February (Fig. 5b). In March, surface activity was detected every day (Fig. 5c). Combining all 3 months with ice cover, the daily estimated rate of surfacing was ~12.5 times fish^−1^. Several of the fish just under the ice repeatedly ascended to the surface before descending (see echogram example Fig. 7), which was a behavior that was not observed in ice-free waters. There was a significant relation between the total area backscattering coefficient (s_a_) and number of surfacing fish during the months with ice cover (January, February, and March; Linear regression, *r* ^2^ = 0.08, *F*_1,71_ = 7.3, *P* = 0.0085), but the coefficient of determination was very low.

Gas release was strongly reduced when the fjord was ice covered, and the pattern of nocturnal gas release that was observed in ice-free waters was not apparent. Gas bubbles were sporadically detected, but only at a rate of 2 times fish^−1^ day^−1^ (Fig. 6). More than 60 % of the gas releases recorded during this period (194 in total) were detected during the last 2 days of March (a couple of days before the ice melted; not shown). The reduced amount of gas bubbles was confirmed by the 120 kHz echosounder which covered nearly the whole range of the sprat population (0–80 m).

### Cloud cover and moon phase

The weather was relatively cloudy during the whole winter with an average cloud index mostly fluctuating between 5 and 8 (8 is maximum) (not shown). No consistent pattern between frequency of surfacing events and cloud index or moon phase was evident.

### Swimming speed and TS measurements

**Fig. 8:**
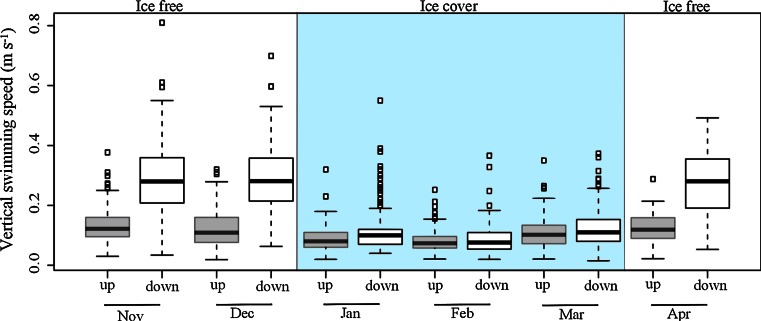
*Box plot* showing vertical swimming speed of ascending and descending sprat when surfacing in November and December 2009 (*n* = 346, *n* = 112, respectively), and January, February, March, and April 2010 (*n* = 324, *n* = 140, *n* = 352, *n* = 46, respectively). *Gray colored boxes* show ascending speed and *white colored* show descending speed. The medians are shown as *horizontal lines*, and the *boxes* are bound by 25 and 75th percentiles

During ice-free conditions, the average downward swimming speed after surfacing was approximately twice as high as the average upward speed, ~28 and ~13 cm s^−1^, respectively (Fig. 8). Vertical swimming associated with surface events was considerably slower when the fjord was ice covered, with the descent and ascent rates being ~11 and ~ 8 cm s^−1^, respectively (Fig. 8). The swimming speeds differed significantly by both swimming direction and environmental conditions (before ice, during ice, and after ice cover), and there was a significant interaction between the two factors (two-way ANOVA, *F*_1,2633_ = 784.6, *F*_2,2633_ = 760.1, *F*_2,2633_ = 342.4, *P* < 0.0000, for all tests). A post hoc Tukey’s test (*P* value of <0.05) revealed that among fifteen possible comparisons, only three were not significantly affected by interaction: downward swimming before ice versus downward swimming after ice (*P* = 0.18), upward swimming before ice versus upward swimming after ice (*P* = 0.99), and downward swimming during ice versus upward swimming after ice (*P* = 0.86). The two factors are difficult to interpret because of the significant interaction; however, the results show that the swimming speed depended on environmental conditions and that the difference between upward and downward swimming speed was greater in ice-free waters (Fig. 8).

The TS values were weaker in ice-covered waters than in ice-free waters. The average TS values of ascending and descending sprat during surfacing in ice-free waters were −54.2 and −53.5 dB, respectively, and −57.8 and −54.3 dB in ice-covered waters, respectively. The TS values differed significantly by both swimming direction and ice conditions (ice-free waters versus ice-covered waters) (two-way ANOVA, *F*_1,2635_ = 100.5, *P* < 0.0000, *F*_1,2635_ = 85.3, *P* < 0.0000), and a significant interaction between the factors was also present here (*F*_1,2635_ = 21.1, *P* < 0.0000). TS of upward swimming sprat in ice-free waters versus TS of downward swimming sprat in ice-covered waters was the one comparison (among six) that was not affected by interaction (*P* = 0.98, post hoc Tukey’s test, *P* < 0.05).

## Discussion

### Patterns of surfacing

The surfacing events were recorded exclusively at night, even when the population as a whole displayed inverse vertical migrations, with an overall deeper nocturnal distribution. Sprat in Bunnefjorden are vulnerable to predation by gadoid fishes when in shallow waters at night (Kaartvedt et al. 2009). The results on swimming speed can be interpreted in this context as the vertical speed when surfacing was ~10 times as high as during normal DVM (populations speeds derived from echograms). The nocturnal pattern of surfacing events indicates that the surfacing behavior is correlated with reduced light level and subsequently reduced risk of predation.

### Surfacing behavior interpreted as air-gulping behavior

Surfacing behavior in sprat has been reported earlier (Kaartvedt et al. 2009). Since sprat is a physostome fish, this is believed to be connected with gulping of atmospheric air, a behavior previously documented in the physostome herring (Brawn 1962, Blaxter and Batty 1984). In this study, the significant correlation found between surfacing behavior and gas bubble release in ice-free waters supports this interpretation. Furthermore, the results on TS and swimming speeds are in accordance with an assumed gulping behavior. Air in the swim bladder accounts for about 90 % of the target strength of a fish (Foote 1980 *b*), and the TS values of descending sprat in ice-free waters were significantly stronger than the TS of descending sprat in ice-covered waters. This was the case in spite of much faster diving (~28 and ~11 cm^−1^, respectively), which might have caused a steeper tilt angle, and thus rather expectedly reduced TS (Røstad 2006). The high descent rate after assumingly gulping air at the surface may be explained by the need to generate extra thrust to counteract positive buoyancy, corresponding to findings for seals and sea birds which are known to strike heavily during the initiation of a dive in order to overcome the hydrostatic force (Sato et al. 2003; Lovvorn et al. 2004; Watanuki et al. 2006). The fact that downward speed was reduced when access to atmospheric air was blocked further supports this assumption. Also, the difference in speed between ascent (relatively slow) and descent (fast) was considerably larger in ice-free waters.

### Surface activity during ice covering

The study site became ice covered in mid-winter, likely blocking access to atmospheric air. Yet, surfacing tracks were frequently recorded, but differed from those in ice-free waters by being characterized by repeated short ascents just under the ice before descent back to deeper waters. Similar behavior was reported in a study of submerged Atlantic salmon (Korsøen et al. 2012) who observed a highly increased swimming activity near the roof of a sea cage in periods without air access; the increased activity was interpreted as “searching for the surface.”

As air bubbles can be trapped in pockets during ice formation, and gas may accumulate under the ice from photosynthesis and anaerobic decomposition, there might have been an alternate source of gas underneath the ice cover. In relation to aerial respiration, Klinger (1978) and Klinger et al. (1982) found that the central mud-minnow (*Umbra limi*) (facultative air-breather and physostome fish) used oxygen directly from gas bubbles found under the ice to prolong its survival in a hypoxic lake. Similar to the mud-minnow, the sprat may have searched for trapped gas underneath the ice. As we found a significant relation between surfacing and gas release in ice-free waters, the latter might be used as indication of how “successful” under-ice surfacing (inferred as under-ice gulping) was (see below).

### Gas bubble release

In ice-free waters, release of gas was observed some hours after surfacing. Gas release in herring has been documented in previous studies (Thorne and Thomas 1990; Suuronen et al. 1997), and whether the source of gas comes from atmospheric air or as a cause of bacterial activity in the digestive duct has been debated (Brawn 1962; Nero et al. 2004). The temporal pattern of surfacing with subsequent air release observed in our study indicates that the gas release is connected to the surfacing behavior. The fact that few gas bubbles were detected when the fjord was ice covered in spite of high surface activity furthermore supports this conclusion.

Thorne and Thomas (1990) documented gas bubble release in Pacific herring during upward vertical migration with a widespread release at shallower depths, possibly triggered by reduced static pressure. Air release in herring as a response to reduced ambient pressure has also been shown in laboratory studies (Brawn 1962; Wahlberg and Westerberg 2003). Such relations were not a consistent pattern in our study. Early in the study period when the sprat carried out IDVM and migrated to shallower waters during dawn, echograms showed that gas bubbles were released in connection with (yet not exclusively related to) upward migration (results not presented). Yet, as the nocturnal distribution became shallower by the end of November, records of surfacing events and gas release became more evenly dispersed throughout the evening and the night, and fish released gas in shallow waters throughout the night without any relation to vertical migrations.

Brawn (1969) suggested that herring released air to make downward swimming easier. We observed gas release prior to sunrise and descent when the sprat carried out normal DVM, but this explanation does not comply with the assumption that clupeids use atmospheric air to maintain buoyancy (Thorne and Thomas 1990).

Several authors have debated whether air release in physostomes is a physical response or a behavioral response. Wilson et al. (2004) and Hahn and Thomas (2008) postulated that gas release not solely serves as a mechanistic adjustment for the swim bladder volume, but may also be related to distress, or serve purposes like predator avoidance and intraspecific communication. Nøttestad (1998) observed air release within herring schools during predator attacks and suggested that gas bubbles may contribute to predator avoidance by scattering the light and reducing the visual range of the predators. Air release in our study was mostly detected during darkness when the fish was not schooling and the risk of predation is reduced. Ascending and descending schools releasing gas bubbles at daytime were, also in our study, sporadically detected by the deeper located echosounder, but with no apparent relation to the presence of predators. Gas release as an anti-predator response is thus not supported by the present observations.

### Importance of air gulping

Sprat ascended to the surface on a daily basis when having access to the surface (the average rate of surfacing was ~3.5 times sprat^−1^ day^−1^). Noticeably, during some days in January when the fjord was ice covered, the average rate of surfacing increased to ~26 times fish^−1^ day^−1^, and combining all months with ice cover, the surfacing rate was ~12.5 times fish^−1^ day^−1^. Negligible amount of observed gas bubbles during this period indicates an unsuccessful outcome in terms of obtaining air. Yet, the consistent pattern of repetitive trials underneath the ice suggests that atmospheric air is an important element in the life strategy of sprat. Fish with negative buoyancy may compensate by tilted swimming (Huse and Ona 1996; Strand et al. 2005), “rise and glide” swimming (Huse and Ona 1996), or by increased swimming speeds (Dempster et al. 2009). Producing additional lift by swimming is, however, energetically costly. Speers-Roesch et al. (2004) found a 20 % higher oxygen uptake in the bloater (*Coregonus hoyi*) when exposed to increased pressure (four atmospheres) due to enhanced swimming activity. Some fish may use oxygen in the swim bladder for respiration during hypoxic conditions, but it is unclear to what extent this also relates to sprat (Kaartvedt et al. 2009). In this study, we found no evidence of change in the frequency of surfacing events after the fjord apparently became more oxygenated from mid-February.

Several studies of salmonids (physostomous fish) have shown that denial of surface access leads to negative buoyancy and may cause increased mortality, reduced welfare (Fosseidengen et al. 1982; Ablett et al. 1989), and deformations in the vertebrae due to tilted “tail-down,” “head-up” swimming (Korsøen et al. 2009). Correspondingly, the particularly high rate of surfacing observed in ice-covered waters suggests that denied surface access may represent a constraint to the sprat. The abundance of sprat generally declined after the fjord froze over (this study, Solberg et al. 2012), yet some evidently remained in Bunnefjorden. The sprat therefore appears to be able to deal with the challenges imposed by the ice cover at costs which remain to be established.

In conclusion, this study has revealed that the sprat regularly carried out rapid excursions to the surface, apparently to gulp air. Hampered access to the surface by ice cover affected both surfacing frequency and swimming behavior, and ice covering may constrain physostome fish in yet unknown ways. In a broader setting, reduced ice coverage will be one effect of global warming, with consequent altered access to the surface for physostome fish. Also, adult physostomes are reported to be highly vulnerable to exposure of surface toxins (such as related to oil spills) because of their migrations to the surface (Thorne and Thomas 2008). Therefore, understanding the importance of surfacing and subsequently air gulping in the life of physostome fish is pertinent also in the context of anthropogenic impact on the environment.
